# Renal medullary perfusion differs from that in renal cortex in patients with sepsis associated acute kidney injury and correlates with renal function prognosis: A prospective cohort study

**DOI:** 10.3233/CH-242296

**Published:** 2024-10-18

**Authors:** Rongping Chen, Dawei Liu, Hua Zhao, Xiaoting Wang

**Affiliations:** Peking Union Medical College Hospital, Beijing, China

**Keywords:** Contrast-enhanced ultrasound, renal cortex, renal medulla, sepsis, acute kidney injury, prognosis

## Abstract

**BACKGROUND:**

Renal perfusion status remains poorly studied at the bedside during sepsis associated acute kidney injury (AKI). The aim of the study is to examine renal cortical and medullary perfusion using renal contrast enhanced ultrasound (CEUS) in septic patients.

**METHODS:**

In this single-center, prospective longitudinal study, septic patients were enrolled. Renal ultrasonography was performed within 24 hours of ICU admission (D1), then repeated at D3, D5 and D7. Each measurement consisted of three destruction replenishment sequences that were recorded for delayed analysis with dedicated software (Vuebox). Renal cortex and medulla perfusion were quantified by measuring time to peak (TTP). Receiver operating characteristic (ROC) analysis was used to evaluate 28-day renal prognosis.

**RESULTS:**

The study included 149 septic patients, including 70 non-AKI patients and 79 AKI patients. Both renal cortical and medullary TTP was longer in the AKI group than in the non-AKI group. The difference of TTP between renal cortex and medulla in AKI group was higher than that in the non-AKI group (*p* = 0.000). Medullary TTP on day 3 had the best performance in predicting the prognosis of 28-day renal function (AUC 0.673, 95% confidence interval 0.528–0.818, *p* = 0.024), and its cut-off value was 45 s with a sensitivity 52.2% and a specificity of 82.1%. Cortical TTP on day 3 also had the performance in predicting the prognosis of 28-day renal function (AUC 0.657, 95% confidence interval 0.514–0.800, *p* = 0.039), and its cut-off value was 33 s with a sensitivity 78.3% and a specificity of 55.0%.

**CONCLUSION:**

Renal medullary perfusion alterations differ from those in cortex, with the medulla is worse. Simultaneous and dynamic assessment of cortical and medullary microcirculatory flow alterations necessary. TTP on day 3, especially medullary TTP, seems to be a relatively stable and useful indicator, which correlates with 28-day renal function prognosis in septic patients. Early correction of renal cortical and medullary perfusion alterations reduces the incidence of adverse renal events.

## Abbreviations

AKI: Acute kidney injury
ANOVA: Analysis of variance
APACHE II: Acute physiology and chronic health evaluation II score
AS: Ascending slope
AT: Arrival time
AUC: Area under the curve
CEUS: Contrast enhanced ultrasound
CKD: Chronic kidney disease
COPD: Chronic obstructive pulmonary disease
CVP: Central venous pressure
DBP: Diastolic blood pressure
DT/2: Descending time/2
DS: Descending slope
EDV: End diastolic velocity
ICU: Intensive care unit
IQR: Interquartile range
HR: Heart rate
KDIGO: Kidney disease improving global outcomes
LVOT VTI: Velocity-time integral of the left ventricular outflow tract
MAP: Mean arterial pressure
MAPSE: Mitral annular plane systolic excursion
MI: Mechanical index
MIMIC-III: Multiparameter intelligent monitoring in intensive care
mTT: Mean transit time
NE: Norepinephrine
PEEP: Positive end-expiratory pressure
PI: Peak intensity
P _(*V*-*A*)_ CO_2_: Central venous-to-arterial carbon dioxide difference
PRVF: Proximal renal venous flow
PSV: Peak systolic velocity
RAP: Right atrial pressure
ROC: Receiver operating characteristic
ROI: Region of interest
RRI: Renal resistance index
RRT: Renal replacement therapy
RT: Rise time
RVSI: Renal venous stasis index
SA-AKI: Sepsis-associated acute kidney injury
SCr: Serum creatinine
ScvO_2_: Central venous oxygen saturation
SD: Standard deviation
SNS: Sympathetic nervous system
SOFA: Sequential organ failure assessment
TAPSE: Tricuspid annular plane systolic excursion
TAV: Time-averaged velocity
TIC: Time-intensity curves
TTP: Time to peak

## Keypoints

1. Decreased renal microcirculatory perfusion is present in septic AKI patients;
2. Renal medullary perfusion decreases more dramatically than renal cortex;
3. Renal microcirculatory perfusion decline in septic AKI patients and renal function prognosis.

## 1 Introduction

Acute kidney injury (AKI) is a common complication of sepsis, occurring in up to 40%–50% of hospitalized patients [1]. The pathophysiologic mechanisms underlying sepsis-associated AKI (SA-AKI) are multifaceted, including inflammation, alterations in renal perfusion and changes in cellular bioenergetics, with alterations in renal blood flow perfusion being key to its development and prognosis [2–4]. The abnormal renal perfusion is crucial in its pathophysiological process, which involves alterations in total renal blood flow and microcirculation [5].

Contrast-enhanced ultrasound (CEUS) is an emerging imaging technique in the field of critical illness using highly echogenic but inert microbubbles to delineate areas of micro vessel perfusion within organs [6–8]. It enables the visualization of renal perfusion at various times and across different kidney regions. Administered via infusion, high-frequency ultrasound pulses disrupt the microbubbles, facilitating the generation of time-intensity curves (TIC) during the reperfusion phase [9–12]. CEUS specifically targets the renal cortex and medulla within the region of interest (ROI), extracting parameters of renal microcirculatory perfusion through a perfusion curve model derived from intensity-over-time data. Its application in studying renal microvascular perfusion is well-documented in both animal models and human subjects. The parameters obtained through CEUS demonstrate strong correlation well with the gold standard measures of renal blood flow, such as para-aminohippurate clearance, in studies involving healthy volunteers [13–19]. CEUS is a new vision of microcirculation in the intensive care unit [20]. In patients with septic shock demonstrated that decreased renal cortical perfusion is associated with the severity of AKI [10].

The kidney, requiring high perfusion for optimal glomerular filtration rate (GFR), receives blood predominantly through the cortex (90%), while the medullary blood volume perfuses cortical tissue first, so its proportion is part of the cortical blood volume. This distribution, coupled with intrarenal shunts, predisposes the medulla to hypoxia. Medullary hypoxia, a consequence of altered intrarenal blood flow, is a pivotal factor in AKI development [21–23]. The aim of the current study is to examine renal cortical perfusion and renal medullary perfusion using CEUS and their prognosis value in a larger cohort of patients with sepsis associated AKI and non-AKI.

## 2 Methods

### 2.1 Study design and setting

This study was designed as a single-center prospective study, conducted at Peking Union Medical College Hospital. Serial assessments were made of the renal macro- and microcirculatory state using CEUS and transthoracic echocardiography at 4 time points: Day one (D1) within 24 h of ICU admission, D1 + 48 h (D3), D1 + 96 h (D5) and D1 + 144 h (D7). The enrolled septic patients were assessed on each study day to determine if they remained in sepsis or septic shock. During the study, patients who without AKI within 72 h or transferred from ICU due to improvement of their condition or automatic discharge, were not studied on subsequent study days (D3, D5, or D7). The protocol received approval from the Ethics Committee of Peking Union Medical College Hospital (approval number I-23PJ1284). Written informed consent was obtained from all participants. All procedures adhered to the ethical standards of the local ethics committee on human experimentation and to the Helsinki Declaration of 1975.

### 2.2 Study population

This study encompassed sepsis patients, aged 18 years or older, in accordance with Kidney disease improving global outcomes (KDIGO) criteria between January 1, 2023, and December 30, 2023 [24]. All the measurements were acquired in patients with stable hemodynamics (i.e. MAP≥ 65 mmHg with a stable norepinephrine infusion over 2 h) and had completed 3 hours of bundle resuscitation. Defining AKI biochemically is challenging when the baseline serum creatinine (SCr) is unknown. For patients with known baseline creatinine, AKI was identified using the lowest SCr value from the 12 months preceding admission. If no preadmission creatinine was available, the lowest value the creatinine returned to after resolution of acute illness was used. Failing all the above and if AKI could not be staged by other criteria, the admission creatinine was used as the baseline value [10]. Excluded from the study were patients who (i) exhibited chronic renal dysfunction or other systemic diseases causing renal dysfunction, (ii) had structural kidney abnormalities, (iii) had received a kidney transplantation, (iv) were pregnant [25], (v) were undergoing maintenance dialysis for chronic renal failure, and (vi) contraindication to SonoVue™ (Bracco SpA, Milan, Italy) contrast or if the intent of treatment was primarily palliative [8, 12]. Pulmonary hypertension, a relative contraindication to SonoVue, was measured with transthoracic echo prior to administration of contrast and withheld if the pulmonary artery systolic pressure was > 90 mmHg.

### 2.3 Contrast-enhanced ultrasound

**Fig. 1:**
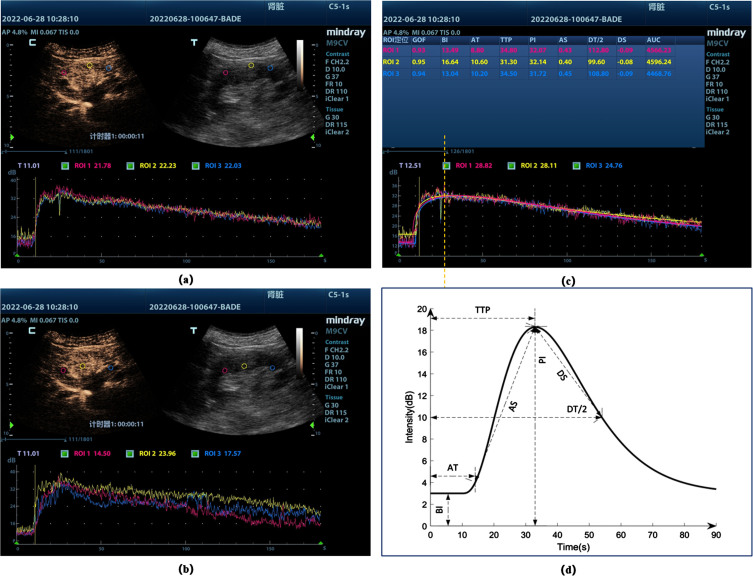
Time-intensity profiles of different sites (a) Cortical sampling site and TIC, the screen shows the contrast-enhanced image (C) As well as the conventional ultrasound image (T); (b) Medullary sampling site and TIC; (c) Plotting of regions of interest (ROIs) analyzed by the software, and then generating the supply curves for each ROI (lower section), these curves represent intensity as a function of time. (d) Schematic diagram of ultrasonography parameters. BI, basic intensity; AT, arrival time; TTP, time to peak; AS, ascending slope; DT/2, descending time/2; DS, descending slope; PI, peak intensity; AUC, area under the curve.

Renal ultrasound was performed using a Mindray SC6-1 s Curved Array transducer has a frequency range from 1.2 MHz. to 5.2 MHz) and SonoVue (Bracco, Italy) as the contrast agent. For CEUS imaging a low mechanical index (MI) was utilized (MI < 0.1) in dual image display format. This format displays two views in parallel; the contrast view is constructed by selectively filtering the signal, identifying microbubbles, while excluding background signal, as a result the initial image is blank prior to the infusion of contrast. The simultaneously displayed standard greyscale image allows the kidney to be identified prior to the infusion (Fig. 1 (a) and (b)). Low MI ultrasound is necessary to avoid microbubble destruction [26]. Once an acceptable and stable view had been established, the intravenous push method involves a single infusion of a specific quantity of contrast agent through a central vein [27]. The specific procedures for CEUS were showed in Table 1. Images of the entire examination were digitally recorded.

**Table 1:** Specific procedures for contrast-enhanced ultrasound

Step	Content
1	Establishment of intravenous access;
2	Select the appropriate probe, abdominal probe is recommended, obtain a standard long-axis view of the kidney, adjust the image depth, focus and frame rate;
3	Entering contrast mode;
4	The contrast agent was added to 5 ml saline according to the instructions and shaken well. The median elbow vein was injected with 0.02 ml/kg of contrast medium and 10 ml of saline washed through the tube;
5	Simultaneous timing and image recording;
6	Sampling sites (regions of interest): three each from the cortex, medulla and corticomedullary junctional zone (avoiding large vessels);
7	Image Acquisition and Analysis;

Post-processing was performed offline using VueBox™ (Bracco Diagnostic Imaging, Switzerland). Cortical regions (Fig. 1. (a)) and medullary regions (Fig. 1 (b)) lying in proximity to the probe with good views and reliably visible reperfusion were identified as ROI. TIC was produced with several key components as shown in Fig. 1(c) and (d). Seven variables were measured from the TIC:

1) Arrival time (AT) — the time post-injection when ROI signal begins to enhance;
2) Time to peak (TTP) — the time post-injection when ROI signal intensity reaches its maximum;
3) Ascending slope (AS) — the slope of ascending part of TIC;
4) Descending time/2 (DT/2) — the half of descending time;
5) Descending slope (DS) — the slope of descending part of the TIC, representing the dilution speed of the ROI;
6) Peak intensity (PI) — the peak intensity is the difference between the maximum and minimum intensity;
7) Area under the curve (AUC) — Area under the TIC curve.

### 2.4 Ultrasonography parameters

Baseline greyscale and color Doppler sonographic images were acquired and the renal resistance index (RRI) measured in an intralobular artery as described elsewhere. This index was calculated as the peak systolic velocity (PSV) minus end diastolic velocity (EDV), divided by the PSV [28]. Color Doppler images facilitated the identification of a target renal vein. The PRVF waveforms, representing flow away from the transducer below the baseline. The RVSI was employed to quantify renal reflux obstruction. This index was calculated as the index cardiac cycle time minus the renal venous flow time, divided by the index cardiac cycle time [29]. At the same time, obtaining baseline cardiac parameters: Velocity-time integral of the left ventricular outflow tract (LVOT VTI); Tricuspid annular plane systolic excursion (TAPSE); Mitral annular plane systolic excursion (MAPSE).

Prior to this study, one investigator, proficient in ultrasound, underwent training sessions to standardize renal ultrasonography procedures. Two sonographers, blinded to all clinical information, reviewed the images and confirmed the final assessment of the TIC of renal cortex and renal medulla. Apart from the study investigators, no other physicians were informed of the renal ultrasonography results.

### 2.5 Other parameters collected

We gathered demographic information (age, gender, height, and weight) along with diagnoses. Additionally, various ICU-related parameters were collected at the time of diagnosis for all patients. These included central venous pressure (CVP), heart rate (HR), systolic blood pressure (SBP), diastolic blood pressure (DBP), mean arterial pressure (MAP), central venous-to-arterial carbon dioxide difference [P _(*V*-*A*)_ CO_2_], central venous oxygen saturation (ScvO_2_), Acute Physiology and Chronic Health Evaluation II score (APACHE II), and Sequential Organ Failure Assessment Score (SOFA).

### 2.6 Outcomes

In patients with ICU-acquired sepsis-associated AKI, we assessed renal recovery at day 28 post-enrollment as the primary outcome. These patients were monitored for 28 days through clinical visits or telephone interviews. Nonrecovery from AKI was defined by either of the following criteria: the last creatinine measurement within the initial 28 days of hospitalization exceeding 1.5 times the baseline value, the need for renal replacement therapy (RRT), or death.

### 2.7 Statistical analysis

Continuous data were assessed for normality using the Shapiro– Wilk test and presented either as median and interquartile range (IQR) or mean and standard deviation (SD), based on the results. These data were then compared using the non-paired *t*-test, one-way ANOVA, Kruskal– Wallis test, or Mann– Whitney U test, depending on their normality. Categorical variables were expressed as numbers and percentages and compared using the Chi-squared test. For variables not following a normal distribution, Spearman’s correlation analysis was utilized. ROC curves were used to evaluate 28-day renal prognosis and 28-day mortality. Statistical analyses were performed using SPSS software version 26.0 (IBM Corp., Armonk, NY, USA) and R software 4.2.2 (R Foundation, Vienna, Austria). All *p*-values were two-sided and considered significant at <0.05.

## 3 Results

**Fig. 2:**
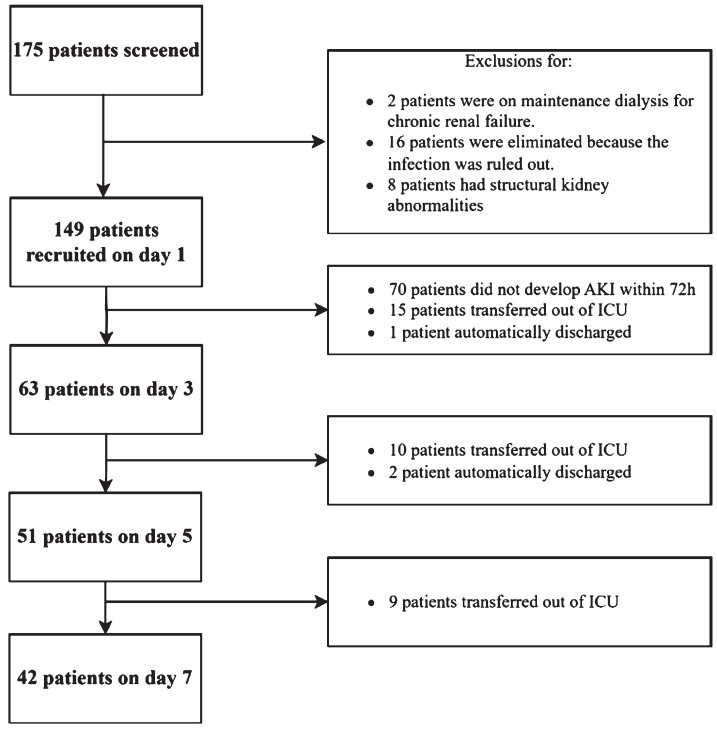
Flow diagram of participants through the study. CEUS, Contrast-enhanced ultrasound; TAPSE, tricuspid annular plane systolic excursion; LVOT VTI, velocity-time integral of the left ventricular outflow tract; MAPSE, mitral annular plane systolic excursion; CVP, central venous pressure; MAP, mean arterial pressure; P*_(*V*-*A*)_* CO_2_, central venous-to-arterial carbon dioxide difference; ScvO_2_, central venous oxygen saturation; AKI, acute kidney injury; ICU, intensive care unit.

As is shown in Fig. 2, 175 sepsis patients were screened during the study period. Out of these, 159 met the inclusion criteria, but 10 were excluded based on the exclusion criteria. Ultimately, 149 patients were enrolled in the study. Provided the sepsis patients with AKI remained stay in the ICU for the study duration, CEUS imaging was achieved in all and at all time points. There were no adverse events from contrast administration. There were 70 patients did not develop AKI within 72 h, while in the sepsis patients with AKI, 15 patients had transferred out of ICU by D3, 10 by D5 and 9 by D7. 1 patient automatically discharged by D3, and 2 by D5. And all completed the 28-day follow-up.

Table 2 presents the demographic characteristics of the study population. The median APACHE II and SOFA were same in the both groups. The median CVP was 8 cmH_2_O in the sepsis associated non-AKI group and 7 cmH_2_O in the SA-AKI group. P_(*V*-*A*)_CO_2_, lactate, and norepinephrine dose were significantly higher in the SA-AKI group than that in the sepsis associated non-AKI, while ScvO_2_ in the SA-AKI group was lower. There are 48.3% of sepsis patients and 51.7% of patients were diagnosis sepsis shock. Among SA-AKI patients, 65.9% of KDIGO 1, 13.9% of KDIGO 2, and 20.3% of KDIGO 3. The median RRI levels were recorded at 0.66 and 0.63 in the SA-AKI group and non-AKI group, respectively. The median RVSI of PRVF levels were recorded at 0.35 and 0.26 in the SA-AKI group and non-AKI group, respectively. There were similar echocardiographic parameters for RBF and both the left and right heart (TAPSE, VTI, MAPSE) in the both groups.

**Table 2:** Demographic characteristics of the study population

	Total (149)	Non-AKI group (70)	AKI group (79)	*p* value
Sex, male, (%)	97 (65.1)	36(51.4)	61(77.0)	
Age (years)	61(49,70)	59(44,70)	62(52,75)	0.108
Height (cm)	170(165,175)	170(165,175)	170(165,173)	0.286
Weight (kg)	68(55,78)	68(55,78)	70(60,80)	0.891
HR (bpm)	87(78,95)	87(73,90)	87(76,95)	0.072
SBP (mmHg)	122(108,132)	118(106,132)	123(111,136)	0.323
DBP (mmHg)	64(57,70)	65(58,72)	63(55,73)	0.299
MAP (mmHg)	82(74,91)	83(77,89)	81(72,92)	0.335
CVP (cmH_2_O)	7(6,9)	8(6,8)	7(6,9)	0.490
P*_(*V*-*A*)_*CO_2_ (mmHg)	4(3,6)	4(3,5)	5(3,7)	0.027*
ScvO_2_ (%)	73(71,77)	75(71,78)	72(70,76)	0.045*
Lactate (mmol/L)	1.6(1.0,2.7)	1.4(0.9,1.8)	2.1(1.1,3.4)	0.005*
APACHE II score	18(14,24)	18(14,22)	18(14,24)	0.910
SOFA score	11(10,13)	11(10,12)	11(10,14)	0.240
Diagnosis, *n*, (%)				
Sepsis	72 (48.3)	51 (72.9)	21 (26.6)	
Sepsis shock	77 (51.7)	19 (27.1)	58 (73.4)	
Infectious source, *n*, (%)				
Intraabdominal	98(65.8)	34(48.6)	64(81)	
Respiratory	34(22.8)	27(38.6)	7(8.9)	
Bloodstream infection	12(8.1)	5(7.1)	7(8.9)	
Musculoskeletal and skin	5(3.4)	4(5.7)	1(1.3)	
AKI KDIGO stage				
1		0	52(65.9)	
2		0	11(13.9)	
3		0	16(20.3)	
Past medical history, *n*, (%)				
Hypertension	62(41.6)	24(34.3)	38(48.1)	
Diabetes	32(21.5)	15(29)	17(21.5)	
Cardiovascular disease	42(28.2)	15(21.4)	27(34.2)	
Cerebrovascular disease	11(7.4)	5(7.1)	6(7.6)	
COPD	3(2.0)	1(1.4)	2(2.5)	
Norepinephrine dose (ug/kg/min)	0.11 (0.00,0.28)	0.071(0.03,0.18)	0.159(0.06,0.39)	0.000*
PEEP (cmH_2_O)	6(5,8)	6(4,10)	6(5,8)	0.323
Time of MV(h)	73(38,211)	60(47,246)	80(27,176)	0.421
Length of ICU days (days)	7(4,10)	8(4,11)	6(4,11)	0.833
Ultrasound parameters				
LVOT VTI (cm)	16.13(14.99,18.78)	16.62(15.13,18.97)	15.9(15.0,18.5)	0.512
MAPSE (cm)	1.15(1.09,1.28)	1.15(1.09,1.31)	1.15(1.03,1.27)	0.733
TAPSE (cm)	1.62(1.45,1.71)	1.59(1.43,1.68)	1.63(1.50,1.78)	0.086
Renal resistance index	0.65(0.60,0.71)	0.63(0.59,0.69)	0.66(0.60, 0.72)	0.008*
RVSI of PRVF	0.30(0.12,0.42)	0.22(0,0.36)	0.35(0.18,0.47)	0.002*

Values are expressed as means±standard deviation, or median (interquartile range), or number (percentage) according to normality test. HR, heart rate; SBP, systolic blood pressure; DBP, diastolic blood pressure; MAP, mean arterial pressure; CVP, central venous pressure; P _(V-A)_ CO_2_, central venous-to-arterial carbon dioxide difference; ScvO_2_, central venous oxygen saturation; APACHE II, Acute Physiology and Chronic Health Evaluation II score; SOFA, Sequential Organ Failure Assessment Score; AKI, acute kidney injury; KDIGO, Kidney Disease Improving Global Outcomes; COPD, chronic obstructive pulmonary disease; PEEP, positive end-expiratory pressure ventilation; MV, mechanical ventilation; ICU, intensive care unit; TAPSE, tricuspid annular plane systolic excursion; LVOT VTI, velocity-time integral of the left ventricular outflow tract; MAPSE, mitral annular plane systolic excursion; RVSI, renal venous stasis index, PRVF, proximal renal venous flow.

Table 3 presents CEUS parameters of the cortex and medulla for septic non-AKI group and AKI group on day 1 and the trend of renal hemodynamic parameters over time in patients in the AKI group. RRI and RVSI were higher in the septic AKI group than in the non-AKI group. Renal cortical AT and renal medullary AT were shorter in the sepsis non-AKI group than in the AKI group. Renal cortical TTP (23.9 vs 31.8 s) and renal medullary TTP (26.5 vs 39.3 s) were shorter in the septic non-AKI group than in the AKI group. Renal cortical and medullary PI and AUC were higher in the AKI group than in the non-AKI group. Trends of renal hemodynamic parameters over time were compared among patients in the AKI group. All renal cortical parameters were not significantly different over time. Whereas renal medullary TTP was shorter on day 7 than on day 1, there was no significant difference in the rest of the parameters over time.

**Table 3:** Comparison of CEUS parameters on day 1 between the non-AKI group and AKI group, as well as between renal cortex and renal medulla over time

		Non-AKI group	AKI group				
		D1(70)	D1(79)	D3(63)	D5(51)	D7(42)	*p*
AT (sec)	Renal cortex	9.37(7.33,11.07)	12.8(9.1,17.0)	12.9(9.5,16.9)	12.1(9.5,16.8)	12.8(9.2,19.0)	0.000*
	Renal medulla	9.63(7.60,12.97)	14.0(9.9, 18.4)	14.3(10.1,19.5)	13.7(10.5,18.4)	13.7(10.0,21.0)	0.000*
	TTP (sec)	Renal cortex	23.9(20.0,28.5)	31.8(26.2,41.8)	34.3(28.73,41.73)	32.0(26.44,45.11)	33.6(24.8,42.4)	0.000*
	Renal medulla	26.5(22.1,31.9)	39.3(31.4,47.5)	40.5(35.24,46.28)	36.0(31.24,41.32)	34.9(31.8,38.4)#	0.000*
AS (dB/s)	Renal cortex	0.48(0.40,0.56)	0.49(0.37,0.71)	0.50(0.40,0.56)	0.54(0.39,0.64)	0.53(0.46,0.64)	0.518
	Renal medulla	0.47(0.40,0.53)	0.39(0.30,0.52)	0.41(0.34,0.47)	0.46(0.34,0.53)	0.47(0.39,0.55)	0.004*
DS (dB/s)	Renal cortex	–0.1(–0.11, –0.08)	–0.10(–0.12, –0.08)	–0.10(–0.12, –0.08)	–0.10(–0.13, –0.08)	–0.11(–0.14, –0.09)	0.179
	Renal medulla	–0.1(–0.11, –0.08)	–0.09(–0.11, –0.07)	–0.08(–0.10, –0.07)	–0.10(–0.13, –0.08)	–0.10(–0.11, –0.09)	0.458
DT/2 (sec)	Renal cortex	109.9(90.5,141.1)	103.7(88.1,130.2)	108.2(91.2,130.0)	107.8(90.7,126.6)	108.78(92.0,124.4)	0.152
	Renal medulla	115.5(90.6,145.4)	106(93.3,135.7)	113.4(97.3,146.1)	105.6(86.1,134.2)	114.7(100.6,130.8)	0.272
PI (dB)	Renal cortex	33.30±3.81	31.77±4.24	32.66±4.67	32.38±5.44	32.73±5.49	0.004*
	Renal medulla	33.30±3.88	30.27±4.27	30.74±4.97	30.89±5.32	31.92±5.88	0.000*
AUC (dB/s)	Renal cortex	6369(4911,7432)	5448(4226,6901)	5999(4595,7305)	5263(4360,7172)	4962(4124,7392)	0.030*
	Renal medulla	6466(4842,7764)	5379(4096,6901)	5790 (4287,8547)	5328(4060,6697)	5119(4298,7055)	0.020*

Values are expressed as means±standard deviation or median (interquartile range). *Denotes significance (*P* < 0.05) between non-AKI group and AKI group. #denotes significance between day 1 and day 7. CEUS, Contrast-enhanced ultrasound; AKI, acute kidney injury; AT, arrival time; TTP, time to peak; AS, ascending slope; DT/2, descending time/2; DS, descending slope; PI, peak intensity; AUC, area under the curve. D1 day 1, D3 day 3, D5 day 5, D7 day 7.

**Fig. 3:**
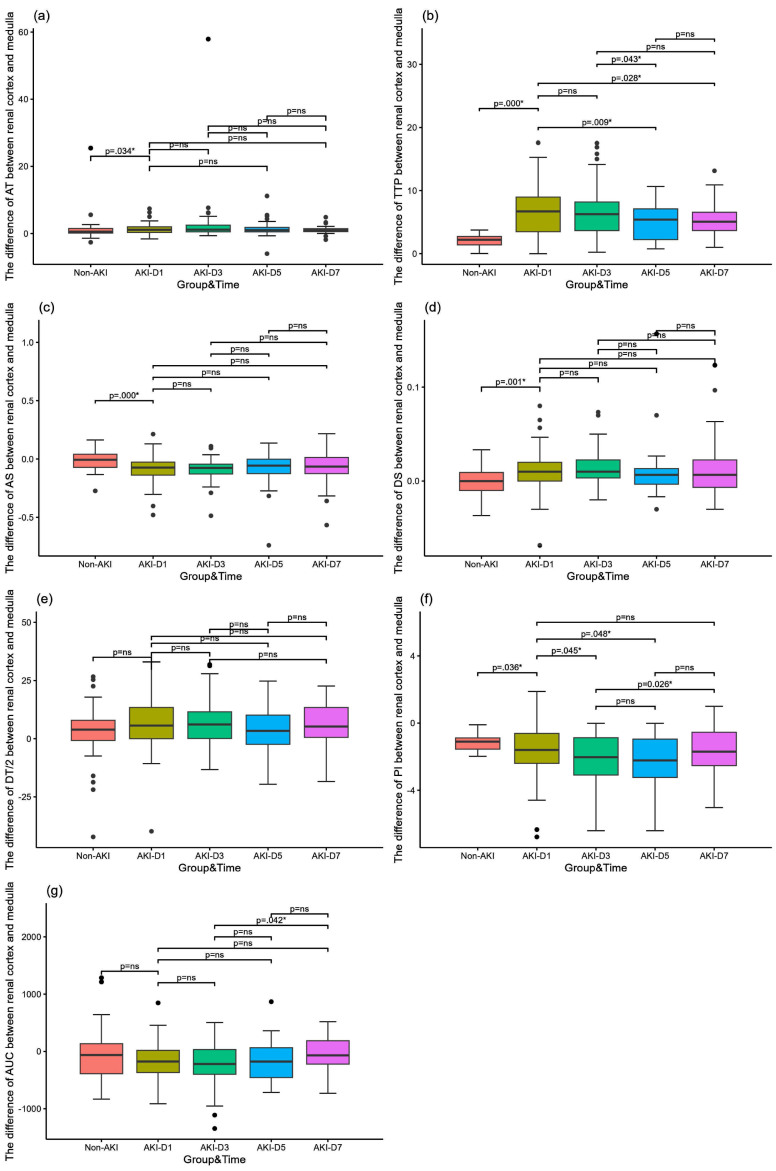
Comparison of the difference in microcirculatory perfusion parameters between the renal cortex and the renal medulla in patients with sepsis. (a) The difference of arrive time (AT) between the AKI group and the non-AKI group and comparison of the AKI group over time; (b) The difference of time to peak (TTP) between the AKI group and the non-AKI group and comparison of the AKI group over time; (c) The difference of slope of rise (AS) between the AKI group and the non-AKI group and comparison of the AKI group over time; (d) The difference of slope of descent (DS) between the AKI group and the non-AKI group and comparison of the AKI group over time; d. Differences in slope of descent (DS) between sepsis AKI and non-AKI groups and comparison of AKI groups over time; (e) The difference of time to descent of dermatomyelin (DT/2) between the AKI group and the non-AKI group and comparison of the AKI group over time; (f) The difference of peak intensity (PI) between the AKI group and the non-AKI group and comparison of the AKI group over time; (g) The difference of area under the curve (AUC) between the AKI group and the non-AKI group and comparison of the AKI group over time.

Table 4 and Fig. 3 show a comparison of the difference between renal cortical and medullary parameters in the AKI and non-AKI groups, as well as a comparison of renal hemodynamic parameters over time in the AKI group. All differences were calculated as renal medullary parameters minus renal cortical parameters. Comparison of the difference between renal cortical and medullary parameters in septic AKI and non-AKI groups showed that the difference in corticomedullary TTP in septic AKI group was significantly higher than that in non-AKI group (*p* = 0.000), and the difference in corticomedullary TTP was significantly lower on day 5 (*p* = 0.009) and day 7 (*p* = 0.028) than that on day 1, and on day 5 than that on day 3 (*p* = 0.043); there was no significant comparison between the two groups for the other time points There was no significant difference between the two groups at other time points. The difference in dermatomedullary PI was significantly lower in the septic AKI group than in the non-AKI group (*p* = 0.036). On day 3 (*p* = 0.045) and day 5 (*p* = 0.048) were significantly lower than on day 1. There was no significant difference between the two at other time points.

**Table 4:** Comparison of the difference in renal cortical and medullary parameters between AKI and non-AKI groups and over time in the AKI group

	Non-AKI group	AKI group				
	D1(70)	D1(79)	D3(63)	D5(51)	D7(42)	*p*
ΔAT (s)	0.6(0.1,1.5)	1.1(0.3,2.0)	1.3(0.5,2.4)	1.0(0.4,1.8)	0.9(0.6,1.4)	0.034*
ΔTTP (s)	2.2(1.4,2.7)	6.7(3.5,9.0) ^bc^	6.3(3.6,8.2) d	5.4(2.2,7.3)	5.1(3.7,6.6)	0.000*
ΔAS (dB/s)	–0.01(–0.07, 0.04)	–0.07(–0.14, –0.03)	–0.08(–0.13, –0.04)	–0.05(–0.12,0.00)	–0.05(–0.12,0.05)	0.000*
ΔDS (dB/s)	0.00(–0.01,0.01)	0.01(0.00,0.02)	0.01(0.00,0.02)	0.01(–0.00,0.01)	0.00(–0.01,0.02)	0.001*
ΔDT/2 (s)	3.9(–1.2,8.4)	5.7(0.0,13.4)	6.2(–0.4,11.6)	4.7(–1.2,13.3)	5.6(1.0,12.7)	0.157
ΔPI (dB)	–1.1(–1.6, –0.9)	–1.6(–2.5, –0.6) ^ab^	–2.2(–3.2, –0.9)	–2.2(–3.7, –0.8)	–1.2(–2.9, –0.3)	0.036*
ΔAUC (dB/s)	–87.9(–411.9,134.9)	–175.9(–369.0,18.4)	–219.0(–411.9, –9.6)	–153.2(–432.1,181.7)	–7.2(–204.3,303.1)	0.138

Values are expressed as means±standard deviation or median (interquartile range). Δ denotes the difference of renal medullary parameter minus renal cortical parameter. *Denotes significance (*P* < 0.05) between non-AKI group and AKI group on day 1. *^*a*^*Indicates *p* < 0.05 for comparison of day 1 and day 3 parameters in the AKI group. *^*b*^*Indicates *p* < 0.05 for comparison of day 1 and day 5 parameters in the AKI group. *^*c*^*Indicates *p* < 0.05 for comparison of day 1 and day 7. *^*d*^*Indicates *p* < 0.05 for comparison of day 3 and day 5. CEUS, Contrast-enhanced ultrasound; AKI, acute kidney injury; AT, arrival time; TTP, time to peak; AS, ascending slope; DT/2, descending time/2; DS, descending slope; PI, peak intensity; AUC, area under the curve. D1 day 1, D3 day 3, D5 day 5, D7 day 7.

**Fig. 4:**
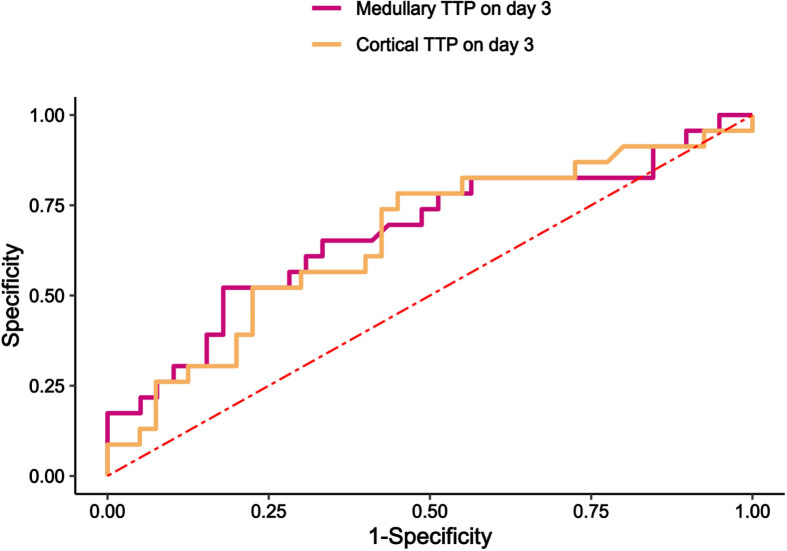
Receiver operating characteristic curves predicated 28-day renal function prognosis. (a) Medullary and cortical TTP on day 3 predicated 28-day renal function prognosis. TTP, time to peak; CEUS, Contrast-enhanced ultrasound.

Table 5 and Fig. 4 present the assessment of CEUS parameters for 28-day renal function prognosis and 28-day mortality. Medullary TTP on day 3 had the best performance in predicting the prognosis of 28-day renal function (AUC 0.673, 95% confidence interval 0.528–0.818, *p* = 0.024), and its cut-off value was 45 s with a sensitivity 52.2% and a specificity of 82.05%. Cortical TTP on day 3 had the performance in predicting the prognosis of 28-day renal function (AUC 0.657, 95% confidence interval 0.514–0.800, *p* = 0.039), and its cut-off value was 33 s with a sensitivity 78.3% and a specificity of 55.0%.

**Table 5:** Area under the receiver operating characteristic curve for CEUS parameters

Indicators	AUC±SE	*P* value	95% *CI*	Cut-off value	Sensitivity	Specificity
Medullary TTP on day 3	0.673	0.024*	0.528–0.818	45s	52.2%	82.1%
Cortical TTP on day 3	0.657	0.039*	0.514–0.800	33s	78.3%	55.0%

Values are expressed as means±standard deviation or median (interquartile range). *Denotes significance (*P* < 0.05). *CI*, confidence interval. AUC, area under the curve; CEUS, Contrast-enhanced ultrasound; TTP, time to peak.

Table 6 shows the comparison of renal outcomes by grouping patients according to the cutoff values for prognosis of renal function assessed in the renal cortex and renal medulla on day 3, respectively. Grouped by cut- off value 33 s of renal cortex, the number of patients with < 33 s was 26, and the number of patients with≥33 s was 37. The number of patients with RRT in the renal cortex TTP≥33 s group was 38.5%, and the duration of RRT and the time to improvement of renal function were longer than in the TTP < 33 s group. Grouped by cut- off value 45 s of renal cortex, the number of patients with < 45 s was 43, and the number of patients with≥45 s was 20. The number of patients with RRT in the renal cortex TTP≥45 s group was 50%, and the duration of RRT and the time to improvement of renal function were longer than those in the TTP < 45 s group.

**Table 6:** Comparison of renal outcome indicators in Subgroups of prognostic cutoff values for assessment of renal function by TTP

	Renal cortex			Renal medulla		
	TTP < 33 s (26)	TTP≥33 s (37)	*p*	TTP < 45 s (43)	TTP≥45 s (20)	*p*
Treated with RRT (%)	4(13.8)	15(38.5)		9(19.1)	10(50.0)	
The duration time of RRT (days)	0(0,0)	0(0,4)	0.027*	0(0,0.5)	1(0,5.5)	0.049*
Renal function improvement time (days)	3(2,9.5)	7(2,28)	0.038*	4(2,11)	28(3,28)	0.010*

Values are expressed as median (interquartile range). *Denotes significance (*P* < 0.05). *CI*, confidence interval. CEUS, Contrast-enhanced ultrasound; TTP, time to peak; RRT, renal replacement therapy.

## 4 Discussion

This is the study to investigate the relationship between renal cortical and medullary blood flow simultaneously in septic patients. It was found that both renal cortical and renal medullary perfusion were decreased in patients with septic AKI, and the decrease was more pronounced in the renal medulla compared to the renal cortex, with the presence of corticomedullary perfusion dissociation. Medullary TTP on day 3 had the highest prognostic performance in assessing 28-day renal function. Comparison of renal outcome indicators by subgroups of TTP cut-off values revealed worse renal outcome indicators in the TTP prolongation group.

Our findings suggested that compared to sepsis associated non-AKI patients, SA-AKI patients decreased renal cortical and medullary perfusion as evidenced by prolonged perfusion time and decreased perfusion intensity. Watchorn et al. showed that decreased renal cortical perfusion in septic shock patients, and correlated with the degree of AKI [30]. Renal cortical perfusion was reduced in patients with septic shock compared to patients without septic shock in ICU, and the reduction in renal cortical perfusion was greatest in the first 24 hours and improved gradually over the next 3 days [31]. Meta-analysis suggested that mean transit time (mTT) and PI were higher compared to the non-AKI group, and a reduced rising slope in the renal cortex in AKI group [32]. Perfusion alterations in the renal cortex in our study were similar to previous studies. There are fewer studies on CEUS assessment of renal medullary perfusion alterations in sepsis patients. Redistribution of intrarenal perfusion, resulting in renal medullary hypoxia, is believed to be a central factor in SA-AKI [33]. The assessment of renal medullary perfusion in SA-AKI patients is essential. Our study found that in comparison to sepsis associated non-AKI patients, renal medullary perfusion also decreased in SA-AKI patients. Is there a consistency between renal cortical and medullary perfusion changes in patients with septic AKI?

In patients with SA-AKI, in contrast to the renal cortex, renal medullary perfusion was decreased and prolongation of perfusion was more pronounced and presence of corticomedullary perfusion differences. The kidney receives blood predominantly through the cortex (90%), while the medullary blood volume perfuses cortical tissue first, so its proportion is part of the cortical blood volume. This distribution, coupled with intrarenal shunts, predisposes the medulla to hypoxia. Medullary hypoxia, a consequence of altered intrarenal blood flow, is a pivotal factor in AKI development [21–23]. In various animal studies, CEUS had shown a consistent pattern of renal perfusion enhancement, initially in the renal cortex and subsequently in the medulla. This pattern enables differentiation between cortical and medullary blood flow in kidneys [34–37]. In healthy cats, cortical PI and mTT were significantly higher than in the medulla [35]. Our study found that in sepsis associated non-AKI patients, CEUS suggested a strong concordance between renal cortical and medullary perfusion, as evidenced by no significant difference in perfusion time and intensity. However, SA-AKI patients showed greater perfusion and shorter perfusion times in the renal cortex than that in the renal medulla. The inter-individual heterogeneity of CEUS measurements should be attributed to the conditions of acquisition in ICU: renal cortical depth based on the patient’s body mass index, respiratory movements that may change the plane of the acquired ultrasound, and changes in tissue thickness and echogenicity over time due to an increase in body fluid balance [38, 39]. In SA-AKI patients, there was a significant difference between renal cortical and renal medullary perfusion, as evidenced by a decrease in medullary perfusion and a more pronounced prolongation of perfusion time. As the kidney is a dual circulatory system, the renal tubular system located in the renal medulla is a low-pressure system and is more susceptible to the elevation of renal backward pressure [40]. Noradrenaline is also known to be a potent venous constrictor, and it is possible noradrenaline contributed to reduced cortical perfusion by increasing the pressure of venous reflux [41]. In our study, both RVSI of PRVF and norepinephrine doses were higher in the SA-AKI group than in the septic non-AKI group. In addition, abnormal intrarenal shunting plays a crucial role in the hemodynamic mechanisms underlying AKI. Enhanced shunting, however, can lead to reduced medullary tissue PO_2_ and varying degrees of tubular hypoxia [42, 43]. Treatment with norepinephrine normalized MAP and increased RBF but further impaired medullary perfusion and oxygenation, suggesting that medullary perfusion is independent of RBF and cortical perfusion [44]. 

Receiver operating characteristic (ROC) curves for 28-day renal prognosis with microhemodynamic data found that medullary TTP on day 3, cortical TTP on day 3, with medullary TTP on day 3 had the highest predictive value. Comparison of renal outcome indicators by subgroups of TTP cut-off values revealed worse renal outcome indicators in the TTP prolongation group. Redistribution of intrarenal perfusion, resulting in renal medullary hypoxia, is an importance factor in sepsis-related AKI [33]. In a sheep model of septic hyperemic renal dysfunction, a reduction in medullary blood flow and oxygen tension was observed before decreases in urine output and creatinine clearance [22]. Prediction of severe AKI during the first 72 h with microhemodynamic data found that mTT on day 0 had the highest predictive value of severe AKI compared to RRI, PI, and AUC [31]. In ICU patients, cortical TTP and medullary rise time may contribute to the diagnosis of AKI [45]. However, the CEUS model used by these investigators relied on continuous infusion administration of contrast and also primary description of the relationship between CEUS and AKI diagnosis, limiting comparisons with the current study. It is evident that alterations in renal medullary blood flow are extremely important for the development and prognosis of AKI. Even though more studies have concluded that the complexity of renal medullary anatomy makes it difficult to assess renal medullary blood flow by CEUS [10]. However, in SA-AKI patients, due to the disparity of renal cortical and renal medullary perfusion alterations, and in conjunction with renal macrocirculation arterial blood flow and renal venous reflux alterations, the hemodynamic etiology leading to blood flow alterations in the renal microcirculation can be rapidly assessed, providing a reliable basis for guiding clinical treatment.

Our findings suggest that among the multiple CEUS parameters, TTP appears to be a relatively stable and useful indicator of prognostic renal function. Although PI also differed between the septic associated AKI and non-AKI groups, PI was heterogeneous, had many influencing factors, and was not found to have a prognostic effect on renal function. Therefore, TTP is an accurate indicator for assessing intrarenal blood flow. There are differences in intrarenal blood flow alterations, with medullary perfusion alterations differing from those in cortex. The next step should be to look for causes of dermatomedullary blood flow separation, especially possible causes that are clinically intervenable, such as elevated renal venous pressure and ischemia at the arterial end of the arterioles, and timely correction of renal microcirculatory perfusion can reduce the incidence of adverse renal complications.

There are several limitations in our study. First, it is a single-center study with a small sample size, and only inclusion of septic patients. While our findings are promising, they require confirmation through further research with a larger cohort. Second, the heterogeneity between individuals should be due to the acquisition conditions in the ICU: the depth of renal cortex according to the patient’s body mass index, the respiratory movements that may modify the acquisition ultrasound plane and the thickness and echogenicity of tissue that are modified over time because of fluid balance increase. This heterogeneity was reported in previous studies of ICU patients; However, our study conducted a cohort study with multiple monitoring dynamics to assess changes in renal perfusion. Some of the results remain inadequately explained, for example, the effect of inconsistent blood flow in the renal cortex and renal medulla, indicating a need for more comprehensive studies in the future. Finally, we report medullary perfusion alterations was different from that of renal cortex, but the measurement of medullary blood flow is inherently difficult due to the complex nature of large vessels in the vicinity of regions with critical perfusion and further experiments are needed to verify our results.

## 5 Conclusions

In conclusion, TTP on day 3, especially medullary TTP, seems to be a relatively stable and useful indicator, which correlates with 28-day renal function prognosis. Early correction of renal cortical and medullary perfusion alterations reduces the incidence of adverse renal events. Renal medullary perfusion alterations differ from those in cortex. Simultaneous and dynamic assessment of cortical and medullary microcirculatory flow alterations necessary.

## Ethics approval and consent to participate

This study was designed as a single-center prospective study conducted at Peking Union Medical College Hospital. The protocol was approved by the Ethics Committee of Peking Union Medical College Hospital (approval number I-23PJ1284). Written informed consents were obtained from all participants.

## Data Availability

The dataset used and analyzed for the current study is available from the corresponding author upon reasonable request.
